# Genome Sequence of *Pseudomonas citronellolis* SJTE-3, an Estrogen- and Polycyclic Aromatic Hydrocarbon-Degrading Bacterium

**DOI:** 10.1128/genomeA.01373-16

**Published:** 2016-12-08

**Authors:** Daning Zheng, Xiuli Wang, Pingping Wang, Wanli Peng, Nannan Ji, Rubing Liang

**Affiliations:** State Key Laboratory of Microbial Metabolism, and School of Life Sciences & Biotechnology, Shanghai Jiaotong University, Shanghai, China

## Abstract

*Pseudomonas citronellolis* SJTE-3, isolated from the active sludge of a wastewater treatment plant in China, can utilize a series of environmental estrogens and estrogen-like toxicants. Here, we report its whole-genome sequence, containing one circular chromosome and one circular plasmid. Genes involved in estrogen biodegradation in this bacterium were predicted.

## GENOME ANNOUNCEMENT

Estrogens are one of the most dangerous environmental endocrine-disrupting compounds, and biodegradation is considered to be the efficient strategy (1–4). Although some estrogen-degrading bacteria have been isolated, the degradation mechanisms vary in different strains and remain poorly characterized (5, 6).

*Pseudomonas citronellolis* SJTE-3 isolated from active sludge of a wastewater treatment plant was capable of utilizing different estrogens (17β-estradiol, estrone, estriol, and 17α-ethynylestradiol), estrogenic chemicals, and some polycyclic aromatic hydrocarbons (PAHs) (naphthalene, bisphenol A, phenanthrene, fluorene, and anthracene) and transforming them into nonestrogenic chemicals (unpublished data). Therefore, strain SJTE-3 may play an important role in the bioremediation of estrogen-polluted environments; its genome sequencing will provide great insights into its genetic variability and assist in the study of its biodegradation mechanisms.

Genomic DNA from *P. citronellolis* SJTE-3 was extracted using the QIAamp DNA minikit (Qiagen, CA), and two libraries were constructed. One was a 10-kb-insert SMRTbell library sequenced on the Pacific Biosciences (PacBio, CA) RSII platform using two single-molecule real-time (SMRT) cells with a single 180-min movie (475,094 reads for 1,488,681,633 bp) using the PacBio RSII sequencer (7). Another one was a 400-bp-insert library, sequenced with paired-end sequencing mode (2 × 251 bp) using Illumina Miseq platform (12,700,872 reads in 3,044,385,325 bp) (8). The gaps were closed by specific PCR and Sanger sequencing. The data of the PacBio RSII platform were assembled using the Celera Assembler and PBjelly software (9, 10), and the data of the Illumina MiSeq platform were corrected with Kmer and assembled with Newbler (11, 12). Finally, all the contigs were emended with Pilon (13). The coding sequences (CDSs) were predicted using Glimmer 3.0 (14); tRNA and rRNA were identified with tRNAscan-SE (15) and RNAmmer (16), respectively. The genome sequence was annotated using the RAST (17) and PGAAP (18) databases. Gene functions were annotated with the NCBI-NR (19), eggNOG (20), Swiss-Prot (21), and KEGG (22) databases. The clustered regularly interspaced short palindromic repeats (CRISPRs) were identified by the CRISPR recognition tool (CRT) (23).

The genome of SJTE-3 contained one circular chromosome and one plasmid, pRBL16, with 67.04% and 56.57% G+C contents, respectively. The coding regions covering 86.21% of the genome (7,309,421 bp) contained 6,756 genes, encoding 6,667 proteins; 199 proteins were encoded by the 370,338-bp plasmid pRBL16. Also, the genome encodes five 5S-16S-23S rRNA operons, 69 tRNAs, 5 noncoding RNAs (ncRNAs), and 2 CRISPR clusters. The predicted proteins were classified into 36 COG categories. The six most abundant subsystems are related to amino acid metabolism (*n* = 817), carbohydrate metabolism (*n* = 546), cofactors, vitamins, and pigments (*n* = 388), protein metabolism (*n* = 300), fatty acids, lipids, and isoprenoids (*n* = 272), and aromatic compound metabolism (*n* = 270). Many CDSs are involved in membrane transport, stress response, motility, chemotaxis, regulation, and cell signaling. According to the proposed degradation pathways (24, 25), several enzymes involved in estrogen degradation were annotated, such as 3-ketosteroid-delta-dehydrogenase, hydroxysteroid dehydrogenase, and Rieske dioxygenase.

In conclusion, the whole-genome sequence of *P. citronellolis* SJTE-3 will enrich the genome database of estrogen degradation microorganism and shed a light on the biodegradation mechanism study of estrogenic chemicals.

### Accession number(s).

This whole-genome shotgun project has been deposited in DDBJ/ENA/GenBank under the accession numbers CP015878 (chromosome) and CP015879 (plasmid pRBL 16). The versions described in this paper are the first versions. The strain is available from the China General Microbiological Culture Collection Center (CGMCC) under the GenBank accession no. 12720.
